# Synthesis and Evaluation of Novel Oxyalkylated Derivatives of 2′,4′-Dihydroxychalcone as Anti-Oomycete Agents against Bronopol Resistant Strains of *Saprolegnia* sp.

**DOI:** 10.3390/ijms17081366

**Published:** 2016-08-22

**Authors:** Susana Flores, Iván Montenegro, Joan Villena, Mauricio Cuellar, Enrique Werner, Patricio Godoy, Alejandro Madrid

**Affiliations:** 1 Departamento de Química, Facultad de Ciencias Naturales y Exactas, Universidad de Playa Ancha, Avda. Leopoldo Carvallo 270, Playa Ancha, Valparaíso 2340000, Chile; s.flore.gonzalez@gmail.com; 2 Escuela de Obstetricia y Puericultura, Facultad de medicina, Campus de la Salud, Universidad de Valparaíso, Angamos 655, Reñaca, Viña del Mar 2520000, Chile; ivan.montenegro@uv.cl; 3 Centro de Investigaciones Biomédicas (CIB), Escuela de Medicina, Universidad de Valparaíso, Av. Hontaneda N° 2664, Valparaíso 2340000, Chile; juan.villena@uv.cl; 4 Facultad de Farmacia, Universidad de Valparaíso, Av. Gran Bretaña N° 1093, Valparaíso 2340000, Chile; mauricio.cuellar@uv.cl; 5 Departamento De Ciencias Básicas, Campus Fernando May Universidad del Biobío, Avda. Andrés Bello s/n casilla 447, Chillán 3780000, Chile; ewerner@ubiobio.cl; 6 Instituto de Microbiología Clínica, Facultad de Medicina, Universidad Austral de Chile, Los Laureles s/n, Isla Teja, Valdivia 5090000, Chile; patricio.godoy@uach.cl

**Keywords:** oxyalkylchalcones, fish pathogen, *Saprolegnia parasitica*, *Saprolegnia australis*

## Abstract

A series of novel oxyalkylchalcones substituted with alkyl groups were designed and synthesized, and the antioomycete activity of the series was evaluated in vitro against *Saprolegnia* strains. All tested *O*-alkylchalcones were synthesized by means of nucleophilic substitution from the natural compound 2′,4′-dihydroxychalcone (**1**) and the respective alkyl bromide. The natural chalcone (**1**) and 10 synthetic oxyalkylchalcones (**2**–**11**) were tested against *Saprolegnia parasitica* and *Saprolegnia australis*. Among synthetic analogs, 2-hydroxy,4-farnesyloxychalcone (**11**) showed the most potent activity against *Saprolegnia* sp., with MIC and MOC values of 125 µg/mL (similar to bronopol at 150 µg/mL) and 175 µg/mL, respectively; however, 2′,4′-dihydroxychalcone (**1**) was the strongest and most active molecule, with MIC and MOC values of 6.25 µg/mL and 12.5 µg/mL.

## 1. Introduction

Oxyprenylated chalcones are an important subclass of naturally occurring chalcones that have been considered merely biosynthetic intermediates of C-prenylated chalcones for decades [1]. Only in the last 10 years have they been characterized as phytochemicals exerting interesting and valuable biological activities [2,3]. In this context, some structure-activity relationship studies have suggested that the antifungal/antimicrobial effects of chalcones are mainly attributable to the presence of phenolic hydroxyl groups, which are known to have high affinity for proteins [4,5,6]; and that the substitution of the chalcone ring system with alkyl groups, especially *O*-alkyl, is thought to increase their lipophilicity, which enhances their antimicrobial activity through interaction with cellular membranes [7]. Based on these premises, the unexplored potential of oxyprenylated chalcones is of great interest; however, low yields of these compounds have traditionally been obtained from natural sources [1,8]. Because of these reasons, research efforts have intensified in chemically synthesizing these compounds, their structural analogs, and their derivatives in recent decades [9,10].

As such, oxyalkylated chalcones present interesting biological properties, being both synthetically and biosynthetically related to flavonoids [11]. These facts motivated us to accomplish the synthesis of novel oxyalkylated derivatives from 2′,4′-dihydroxychalcone (**1**) with different alkyl moieties. In terms of application, our final aim was the evaluation of their biological activity as potential anti-oomycete agents against *S. parasitica* and *S. australis*.

## 2. Results and Discussion

### 2.1. Synthesis

The goal of our synthesis was to introduce an alkyl group connected to ring A in the 4′ position of the chalcone with an ether link. Interestingly, our chalcone derivatives all possessed a hydroxyl group attached to ring A in the 2′ position.

**Scheme 1:**
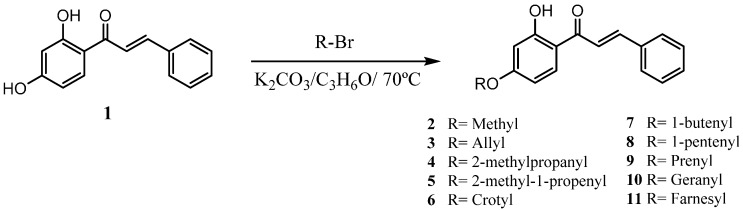
General procedure for the synthesis of *O*-alkylated chalcones.

All of the *O*-alkylated chalcones **2**–**11** were synthesized according to previous literature, i.e., via reaction of 2′,4′-dihydroxychalcone (isolated naturally from *Adesmia balsamica*) with each desired alkyl bromide in the presence of potassium carbonate and acetone (Scheme 1) [12].

Alkyl bromides are a readily available starting material, and, under basic conditions, react with 2′,4′-dihydroxychalcone (**1**), forming high yields of derivatives. The selectivity of the *O*-alkylation at position 4′ of **1** could be explained by the intramolecular hydrogen bond between the carbonyl group and the hydrogen of the hydroxyl group in position 2′ of the aromatic ring A [13]. The hydrogen bond prevents the alkylation of phenol group from occurring in position 2′.

### 2.2. Structure Determination

All compounds have been characterized on the basis of spectral studies (FT-IR, NMR and HRMS data). Assignments of the ^1^H NMR and ^13^C NMR resonances of these compounds were deduced on the basis of signal multiplicities, and by the concerted application of the two-dimensional NMR technique (HMBC).

**Figure 1:**
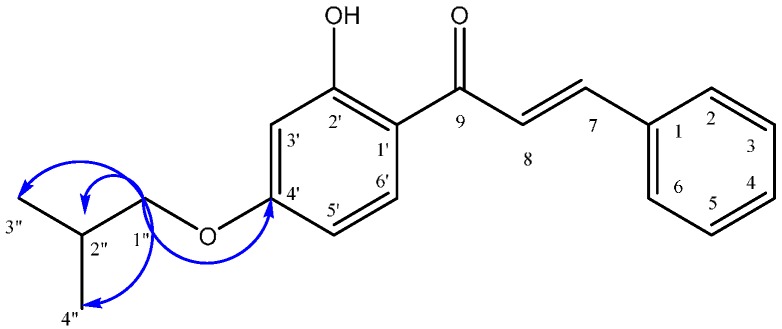
Most important correlations 2D ^1^H–^13^C HMBC, compound **4**.

The ^1^H and ^13^C NMR data for the derivatives of 2′,4′-dihydroxychalcone **2**–**11** were nearly identical in the aromatic region of the spectra. The spectra of these compounds showed two multiplets at δ: 7.66–7.64 ppm and δ: 7.43 ppm integrating for two and three protons, which were assigned to the H-2,6 and H-3,4,5, respectively, pattern characteristic of chalcones with an unsubstituted ring B, and resonances due to the trans-α,β-unsaturated ketone protons at δ 7.65 ppm (1H, *J* = 15.5 Hz, H-7) and 7.57 (1H, *J* = 15.5 Hz, H-8). The ketone carbonyl carbon occurred at δ 191.8–189.9 ppm in the ^13^C NMR spectrum. The ^1^H NMR spectrum of **2**–**11** showed a singlet of a hydrogen-bonded hydroxy proton at δ 13.49–13.40 ppm, confirming the selectivity of the *O*-alkylation at position 4′ of compound **1**. However, alkylation resulted in significant changes in the ^1^H NMR spectra. The spectrum of compound **2** shows the presence of a singlet at 3.88 ppm. This is characteristic of methoxy groups on aromatic rings, resulting from the methyl groups added by the alkylation reaction. For saturated compound **4**, the proton signal at 3.78 ppm (d, 2H) ascribed to O–CH_2_ protons of alkoxy chain linking (*E*)-chalcone ring is also observed. The correlation between the signal at 19.3 and 166.0 ppm with the ^1^H NMR signal cited above allows the assignment of this signal to the O–CH_2_ carbon (Figure 1).

**Figure 2:**
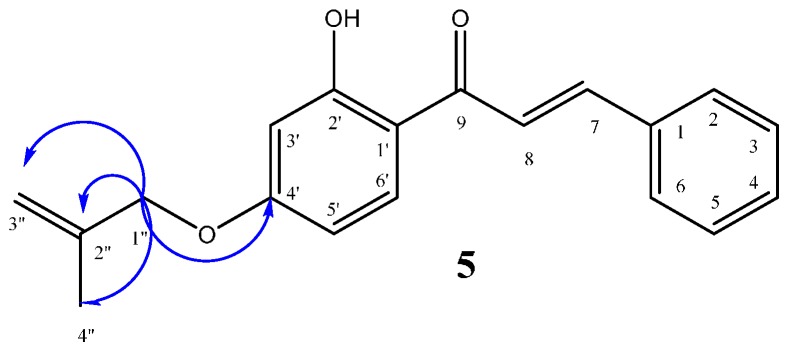
Most important correlations 2D ^1^H–^13^C HMBC, compound **5**.

For allylic compounds **3**, **5**, **6**, **9**, **10** and **11**, the proton signal at 4.47–4.60 ppm (d, 2H) ascribed to O–CH_2_ protons of alkoxy chain linking (*E*)-chalcone ring is also observed. The structural determination of these compounds was established by comparison of the spectral data of compound **4** and using the same criteria. For example, the ^1^H NMR spectrum of compound **5** showed the signal at 4.47 ppm (d, *J* = 6.5 Hz, 2H, H-1″) correlated (by 2D HSQC) with a carbon atom at δ 75.7 ppm (C-1″), indicating the coupling point between the allyl fragment and the aromatic hydroxyl group. These data also were corroborated by 2D HMBC correlations, where H-1″ showed heteronuclear *^3^J* correlations with the carbons signal at δ 19.3 (C-4″), 139.3 (C-3″) and 165.4 (C-4′) ppm. Heteronuclear *^2^J* correlations were also observed at δ 139.9 (C-2″) (Figure 2).

**Figure 3:**
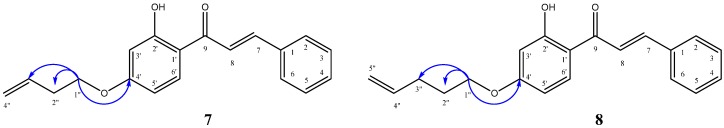
Most important correlations 2D ^1^H–^13^C HMBC, compounds **7** and **8**.

For unsaturated compounds **7** and **8**, the proton signal at 4.08 ppm (t, 2H) ascribed to O–CH_2_ protons of alkoxy chain linking (*E*)-chalcone ring is also observed. For compounds **7**–**8**, the correlation between the signal at 166.7 ppm with the ^1^H NMR signal cited above allows the assignment of this signal to the O–CH_2_ carbon. However, the difference between **7** and **8** was HMBC correlations between H-1″ and C-3″, where H-1″ showed heteronuclear *^3^J* correlations with C-3″ at δ 133.9 and 28.1 ppm, respectively (Figure 3).

### 2.3. Antioomycete Activity against S. parasitica and S. australis in Vitro

In recent years, Bronopol (2-bromo-2-nitropropane-1,3-diol), a broad-spectrum biocide, has seen usage as an effective and economically acceptable alternative in the treatment and control of *Saprolegnia* sp. [14]; however, there are growing concerns regarding its use within the industry, due to its acute toxicity to several fish species and its potentially harmful effects to human health [15]. Furthermore, it has been reported that prolonged use of this product has caused *Saprolegnia* to develop multiresistance mechanisms to the action of this compound [16]. Tests of the antioomycete activities of natural compound **1** and oxyalkylchalcones **2**–**11** were performed, and the results compared to commercial antifungal “Bronopol″; the minimum inhibitory concentrations (MIC) (Table 1) and the minimum oomycidal concentrations (MOC) (Table 2) for **1**–**11** were determined.

Several studies have demonstrated that oomycete fungicide resistance may be linked to specific mutations in target sites [17,18], or other forms of tolerance in some species of oomycete [19]. Further analyses of bronopol resistance in resistant strains of *Saprolegnia* indicates that the former withstood standard concentrations (i.e., regularly used for treating saprolegniasis in farms [17,18]) of the latter. Even though bronopol concentrations used in these studies had a fungistatic effect, and were able to inhibit sporulation, full growth and sporulation ability were recoverable after being cultivated with bronopol at the lesser concentration of 175 (µg/mL) after initial treatment applications.

Table 1 presents the MIC values obtained for isolates of *S. parasitica* and *S. australis*, which, in initial screenings, presented some susceptibility to chalcone **1** and synthetic compounds **2**–**11**. In addition, Table 1 shows comparison values for bronopol, safrole, eugenol, fluconazole and ketoconazole. The MIC values for bronopol varied between 200 and 175 µg/mL for *S. parasitica* and *S. australis*, respectively. In previous studies, azoles (fluconazole and Ketoconazole) had exhibited promising activity, with MIC values of 256 and 64 µg/mL against *Saprolegnia diclina*, *Saprolegnia ferax* and *S. parasitica* [20]. Building on this, our study determined MIC values for fluconazole (>200 µg/mL) and ketoconazole (75 and 50 µg/mL). Another study [21] had shown that safrole and eugenol are effective anti-oomycete agents against *S. parasitica*, *S. diclina*, and *S. ferax*. For compound **1**, the MIC values ranged between 12.5 and 6.25 µg/mL for both species. For compound **5**, the MIC values ranged between 175 and 150 µg/mL. Compound **11** was the most effective synthetic derivative, with MIC values of 150 and 125 µg/mL, respectively. Results for compound **1** suggested that cell wall integrity may be affected. In short, this compound caused 100% damage (see Table 1) to cell integrity, which may be linked to the β-1,3-glucan synthesis axis; this explanation is feasible, since the oomycete family *Saprolegniaceae* (i.e., *Saprolegnia ferex*, *Achlya bisexualis*, and *Achlya ambisexualis*) are unable to adjust hyphal turgor pressure in response to increasing extracellular osmotic potentials, and thus secrete endoglucanases to reduce the strength of their cell walls [22]. Compound **11** presents membrane damage of 30%–32% in *S*. *parasitica* and *S*. *australis*, similar values to those of bronopol. Next, terms of the minimum oomycetidal concentration (MOC), compound **1** presents a value of 12.5 μg/mL for both species of *Saprolegnia*. Compound **7** had an MOC effect at 175 µg/mL, except in *S. parasitica*, where it was more effective. Compound **11** had oomycetidal activity at concentrations of 125 µg/mL for *S. parasitica* and *S. australis.* The corresponding MOC values in traditional anti-oomecyte agents were similar: Bronopol varies between 200 and 175 µg/mL for *S*. *parasitica* and *S*. *australis*. In previous studies, bronopol exhibited promising activity, with MOC value of 20 µg/mL against *S. parasitica* [20]. Indeed, another study showed that bronopol has even better anti-oomycete effects against *Saprolegnia* with MOC range 100 to 200 µg/mL [23]. For safrole, MOC values were at 200 µg/mL for *S. australis.* Mycelial growth inhibition (MIG) on *S. parasitica* and *S. australis* was estimated by measuring the radial growth of the isolate on PDA plates with a concentration of 200 µg/mL for all compounds and bronopol (see Table 3).

The effect of natural compound **1** on sporulation was assessed by exposing mycelial colonies to compound **1** at a concentration of 200 µg/mL for 30 min. The number of zoospores released was calculated after 48 h. Results for **1** present 100% inhibition of growth respectively for both species. The other compounds **2**–**11** and bronopol had less inhibitory effects. Beyond the practical uses of the compounds tested here, and of interest for future studies, a novel characteristic of the study found that chalcones inhibit β(1,3)-glucan and chitin synthases [24]. It has been reported that *Saprolegnia* cells exhibit similar distributions of cellulose and β-(1→3)-glucan in their walls as those found in *Achlya* [25,26]. In concluding, the results obtained in this work reveal that the highest susceptibility of *S. parasitica* and *S. australis* is to natural compound (**1**) when compared to its synthetic derivates **2**–**11**, bronopol, safrole, or eugenol. In general, **1** showed higher inhibition of growth values as compared to positive controls tested. The oxyalkylchalcone **11** tested was more efficient than bronopol, safrole, and eugenol. Among the group of synthetic *O*-alkylated chalcones, the farnesyloxy derivate was a close second in terms of antioomycete activity against *S. parasitica* and *S. australis*. These results demonstrate a correlation between biological activity and alkyl group length, providing further evidence of the potent activity of farnesyl phenyl ether, as had been touched on in previous studies [27,28]. In the event that natural chalcone compound **1** were to replace synthetic compounds, hatchery operating costs would very likely be reduced, due to the low cost with which this compound can be obtained, and workplace safety would be improved. Moreover, the usage of natural 2′,4′-dihydroxychalcone instead of bronopol or formalin would relieve some of the environmental pressures on fish farms, which have been encouraged to pollute less in operations and reduce impacts on the environment in recent years.

## 3. Materials and Methods

### 3.1. General

Unless otherwise stated, all chemical reagents and positive controls purchased (Aldrich, Darmstadt, Germany) were of the highest commercially available purity and were used without previous purification. Structures of synthesized products were confirmed by spectroscopic methods have been given elsewhere [29]. The course of synthesis was controlled by means of thin layer chromatography and products were separated by column chromatography following a standard method [29].

### 3.2. Plant Material

Aerial parts of *Adesmia balsamica* were collected in Viña del Mar, Valparaíso Region, Chile, in March 2015. A voucher specimen (VALPL 1899) was deposited at the VALP Herbarium, Department of Biology, Universidad de Playa Ancha, Valparaíso, Chile.

### 3.3. Isolation of 2,4-Dihydroxychalcone (**1**)

The natural chalcone **1** was isolated from resinous exudate of *A. balsamica* (Fabaceae). The extraction methodology and isolation of pure compound was performed according to reported procedures [30]. Compound **1** was identified by melting point, spectroscopic data, including ^1^H and ^13^C NMR and comparisons with data reported in the literature [31]. The % purity of compound **1** (95%) was confirmed by analytical HPLC.

### 3.4. Synthesis

#### 3.4.1. Oxyalkylation Reaction

In a round bottom flask compound **1** (5 mmol) was added, and different alkyl bromides (6 mmol) and anhydrous potassium carbonate (0.70 g; 5 mmol) in dry acetone (10 mL) were refluxed for 8 h at 70 °C. Then, the mixture was cooled and diluted with water (30 mL) and extracted with ethyl acetate (2 × 20 mL). The organic layer was washed with brine, dried over anhydrous Na_2_SO_4_, filtered and concentrated under vacuum. The products were purified by crystallization from methanol or the crude was redissolved in CH_2_Cl_2_ (5 mL) and chromatographed on silica gel with hexane/ethylacetate mixtures of increasing polarity (20.0:0.0→16.8:3.2).

#### 3.4.2. Synthesis of Oxyalkylated Chalcones

*(2E)-1-(2-Hydroxy-4-methoxyphenyl)-3-phenylprop-2-en-1-one* (**2**). Compound **2** was obtained as a yellow solid (89%) by coupling of compound **1** (5 mmol) and iodomethane (6 mmol) in acetone. NMR data of **2** was consistent with that reported in the literature [32].

*(2E)-1-[4-(Allyloxy)-2-hydroxyphenyl]-3-phenylprop-2-en-1-one* (**3**). The compound **3** was obtained as a yellow solid (79%) by coupling of compound **1** (5 mmol) and allyl bromide (6 mmol) in acetone. NMR data of **3** was consistent with that reported in the literature [33].

*(2E)-1-(2-Hydroxy-4-isobutoxyphenyl)-3-phenylprop-2-en-1-one* (**4**). Compound **4** was obtained as a pale yellow viscous oil (78% yield) by standard nucleophilic substitution reaction of compound **1** (5 mmol) with 1-bromo-2-methylpropane (6 mmol) in acetone. Compound **4**: IR (cm^−1^) 2961, 1632 (C=O), 1521 (C=C), 1245, 1131; 1120, 1023. ^1^H NMR (CDCl_3_, 400.1 MHz) δ 13.36 (s, 1H, 2′-OH), 7.89 (s, 1H, H-7); 7.84 (m, 1H, H-6′); 7.66 (m, 2H, H-2 and H-6); 7.57 (s, 1H, H-8); 7.43 (m, 3H, H-3, H-4 and H-5); 6.47 (m, 2H, H-3′ and H-5′); 3.78 (d, *J* = 6.52 Hz, 2H, H-1″); 2.27 (m, 1H, H-2″); 1.12 (s, 6H, H-3″ and H-4″); ^13^C NMR (CDCl_3_, 100.6 MHz) δ 191.5 (C-9); 166.0 (C-4′); 164.04 (C-2′); 144.1 (C-7); 134.6 (C-1); 131.1 (C-6′); 130.5 (C-4); 129.1 (C-3 and C-5); 128.6 (C-2 and C-6); 121.8 (C-1′); 120.2 (C-8); 107.2 (C-5′); 101.4 (C-3′); 75.7 (C-1″); 28.6 (C-2″); 19.3 (C-3″and C-4″). MS: M + H ion *m*/*z* 297.3671 (calculated for C_19_H_20_O_3_: 296.3604).

(*2E)-1-{2-Hydroxy-4-[(2-methylprop-2-en-1-yl)oxy]phenyl}-3-phenylprop-2-en-1-one* (**5**). The compound **5** was obtained as a yellow oil (72% yield) by standard nucleophilic substitution reaction of compound **1** (5 mmol) with 3-bromo-2-methyl-1-propene (6 mmol) in acetone. Compound **4**: IR (cm^−1^) 2958, 1635 (C=O), 1519 (C=C), 1243, 1131; ^1^H NMR (CDCl_3_, 400.1 MHz) δ 13.40 (s, 1H, 2′-OH), 7.91 (s, 1H, H-7); 7.84 (m, 1H, H-6′); 7.64 (m, 2H, H-2 and H-6); 7.57 (s, 1H, H-8); 7.43 (m, 3H, H-3, H-4 and H-5); 6.51 (m, 2H, H-3′ and H-5′); 5.10 (s, 1H, H-3″b); 5.03 (s, 1H, H-3″α); 4.47 (d, *J* = 11.12 Hz, 2H, H-1″); 1.84 (s, 3H, H-4″); ^13^C NMR (CDCl_3_, 100.6 MHz) δ 191.8 (C-9); 166.6 (C-2′); 165.4 (C-4′); 144.8 (C-7); 139.9 (C-2″); 134.8 (C-1); 131.2 (C-6′); 130.6 (C-4); 129.0 (C-3 and C-5); 128.7 (C-2 and C-6); 122.4 (C-1′); 120.4 (C-8); 113.4 (C-3″); 108.2 (C-5′); 102.0 (C-3′); 71.9 (C-1″); 19.3 (C-4″). MS: M + H ion *m*/*z* 295.3565 (calculated for C_19_H_18_O_3_: 294.3345).

*(2E)-1-[4-(Crotyloxy)-2-hydroxyphenyl]-3-phenylprop-2-en-1-one* (**6**). Compound **6** was obtained as an orange solid (72% yield) by standard nucleophilic substitution reaction of compound **1** (5 mmol) with crotyl bromide (6 mmol) in acetone. Compound **6**: mp 108–110 °C. IR (cm^−1^) 2958, 1635 (C=O), 1519 (C=C), 1243, 1131; ^1^H NMR (CDCl_3_, 400.1 MHz) δ 13.43 (s, 1H, 2′-OH), 7.91 (s, 1H, H-7); 7.84 (m, 1H, H-6′); 7.64 (m, 2H, H-2 and H-6); 7.57 (s, 1H, H-8); 7.43 (m, 3H, H-3, H-4 and H-5); 6.49 (m, 2H, H-3′ and H-5′); 5.93 (m, 1H, H-2″); 5.73 (m, 1H, H-3″); 4.51 (d, *J* = 6.1 Hz, 2H, H-1″); 1.77 (s, 3H, H-4″); ^13^C NMR (CDCl_3_, 100.6 MHz) δ 191.8 (C-9); 166.6 (C-2′); 165.4 (C-4′); 144.8 (C-7); 134.8 (C-1); 131.6 (C-2″); 131.1 (C-6′); 130.6 (C-4); 129.0 (C-3 and C-5); 128.5 (C-2 and C-6); 125.0 (C-1′); 120.4 (C-8); 114.1 (C-3″); 108.3 (C-5′); 101.8 (C-3′); 69.0 (C-1″); 17.9 (C-4″). MS: M + H ion *m*/*z* 295.3512 (calculated for C_19_H_18_O_3_: 294.3444).

(*2E)-1-[4-(But-3-en-1-yloxy)-2-hydroxyphenyl]-3-phenylprop-2-en-1-one* (**7**). The compound **7** was obtained as a yellow oil (79% yield) by standard nucleophilic substitution reaction of compound **1** (5 mmol) with 4-bromo-butene (6 mmol) in acetone. Compound **7**: IR (cm^−1^) 2950, 1636 (C=O), 1522 (C=C), 1245, 1133; ^1^H NMR (CDCl_3_, 400.1 MHz) δ 13.43 (s, 1H, 2′-OH), 7.91 (s, 1H, H-7); 7.84 (m, 1H, H-6′); 7.66 (m, 2H, H-2 and H-6); 7.57 (s, 1H, H-8); 7.43 (m, 3H, H-3, H-4 and H-5); 6.49 (m, 2H, H-3′ and H-5′); 5.90 (s, 1H, H-3″); 5.17 (m, 2H, H-4″); 4.08 (t, *J* = 6.4 Hz, 2H, H-1″); 2.57 (m, 2H, H-2″); ^13^C NMR (CDCl_3_, 100.6 MHz) δ 191.8 (C-9); 166.7 (C-4′); 165.4 (C-2′); 144.4 (C-7); 134.8 (C-1); 133.9 (C-3″); 131.2 (C-6′); 130.6 (C-4); 129.0 (C-3 and C-5); 128.5 (C-2 and C-6); 120.4 (C-8); 117.4 (C-4″); 114.1 (C-1′); 108.1 (C-5′); 101.6 (C-3′); 67.6 (C-1″); 33.3 (C-2″). MS: M + H ion *m*/*z* 295.3514 (calculated for C_19_H_18_O_3_: 294.3445).

*(2E)-1-[2-Hydroxy-4-(pent-4-en-1-yloxy)phenyl]-3-phenylprop-2-en-1-one* (**8**). Compound **8** was obtained as a pale brown oil (78% yield) by standard nucleophilic substitution reaction of compound **1** (5 mmol) with 5-bromo-pentene (6 mmol) in acetone. Compound **8**: IR (cm^−1^) 2958, 1633 (C=O), 1517 (C=C), 1240, 1130; ^1^H NMR (CDCl_3_, 400.1 MHz) δ 13.44 (s, 1H, 2′-OH), 7.91 (s, 1H, H-7); 7.84 (m, 1H, H-6′); 7.65 (m, 2H, H-2 and H-6); 7.57 (s, 1H, H-8); 7.43 (m, 3H, H-3, H-4 and H-5); 6.48 (m, 2H, H-3′ and H-5′); 5.85 (m, 1H, H-4″); 5.06 (m, 2H, H-5″); 4.08 (t, *J* = 6.4 Hz, 2H, H-1″); 2.25 (m, 2H, H-3″); 1.92 (m, 2H, H-2″); ^13^C NMR (CDCl_3_, 100.6 MHz) δ 191.8 (C-9); 166.7 (C-4′); 165.8 (C-2′); 144.3 (C-7); 137.4 (C-4″); 134.8 (C-1); 131.2 (C-6′); 130.6 (C-4); 129.0 (C-3 and C-5); 128.5 (C-2 and C-6); 120.4 (C-8); 115.5 (C-5″); 114.0 (C-1′); 108.1 (C-5′); 101.6 (C-3′); 67.6 (C-1″); 30.0 (C-2″); 28.1 (C-3″). MS: M + H ion *m*/*z* 309.3782 (calculated for C_20_H_20_O_3_: 308.3710).

*(2E)-1-[2-Hydroxy-4-(prenyloxy)phenyl]-3-phenylprop-2-en-1-one* (**9**).The compound **9** was obtained as a yellow solid (70% yield) by standard nucleophilic substitution reaction of compound **1** (5 mmol) with prenyl bromide (6 mmol) in acetone. NMR data of **9** was consistent with that reported in the literature [33].

*(2E)-1-[4-(Geranyloxy)-2-hydroxyphenyl]-3-phenylprop-2-en-1-one* (**10**). The compound **10** was obtained as an orange oil (47% yield) by standard nucleophilic substitution reaction of compound **1** (5 mmol) with geranyl bromide (6 mmol) in acetone. Compound **10**: IR (cm^−1^) 2940, 2865, 1631 (C=O), 1530 (C=C), 1512, 1338, 1241, 1129, 817. ^1^H NMR (CDCl_3_, 400.1 MHz) δ 13.43 (s, 1H, 2′-OH), 7.91 (s, 1H, H-7); 7.84 (d, *J* = 9.6 Hz, 1H, H-6′); 7.65 (m, 2H, H-2 and H-6); 7.57 (s, 1H, H-8); 7.43 (m, 3H, H-3, H-4 and H-5); 6.50 (m, 2H, H-3′ and H-5′); 5.48 (m, 1H, H-2″); 5.09 (m, 1H, H-7″); 4.60 (d, *J* = 6.6 Hz, 2H, H-1″); 2.12 (m, 4H, H-5″ and H-6″), 1.75 (s, 3H, H-4″); 1.68 (s, 3H, H-9″), 1.61 (s, 3H, H-10″). ^13^C NMR (CDCl_3_, 100.6 MHz) δ 191.8 (C-9); 166.7 (C-2′); 166.0 (C-4′); 144.3 (C-7); 141.2 (C-3″); 134.9 (C-1); 131.2 (C-6′ and C-8″); 130.6 (C-4); 129.0 (C-3 and C-5); 128.5 (C-2 and C-6); 123.7 (C-7″); 120.4 (C-8); 118.5 (C-2″); 114.0 (C-1′); 108.3 (C-5′); 101.8 (C-3′); 65.3 (C-1″); 39.5 (C-5″); 26.3 (C-6″); 25.6 (C-10″); 17.7 (C-9″); 16.7 (C-4″). MS: M + H ion *m*/*z* 377.5221 (calculated for C_25_H_28_O_3_: 376.5130).

(*2E)-1-[4-(Farnesyloxy)-2-hydroxyphenyl]-3-phenylprop-2-en-1-one* (**11**). The compound **11** was obtained as a pale yellow oil (31% yield) by standard nucleophilic substitution reaction of compound **1** (5 mmol) with farnesyl bromide (6 mmol) in acetone. Compound **11**: IR (cm^−1^) 2955, 1632 (C=O), 1541 (C=C), 1374, 1203, 1140, 978. ^1^H NMR (CDCl_3_, 400.1 MHz) δ 7.85 (d, *J* = 9.6 Hz, 1H, H-6′); 7.70 (s, 1H, H-7); 7.69 (s, 1H, H-8); 7.58 (m, 2H, H-2 and H-6); 7.35 (m, 3H, H-3, H-4 and H-5); 6.56 (m, 1H, H-5′); 6.52 (m, 1H, H-3′); 5.50 (m, 1H, H-2″); 5.10 (m, 2H, H-7″ and H-12″); 4.60 (d, *J* = 6.6 Hz, 2H, H-1″); 2.07 (m, 4H, H-5″ and H-6″), 1.99 (m, 4H, H-10″ and H-11″), 1.76 (s, 3H, H-4″); 1.73 (s, 3H, H-10″); 1.67 (s, 6H, H-9″ and H-14″). ^13^C NMR (CDCl_3_, 100.6 MHz) δ 189.9 (C-9); 163.7 (C-2′); 160.1 (C-4′); 142.0 (C-7); 142.0 (C-3″); 135.8 (C-8″); 135.6 (C-1); 133.1 (C-6′); 131.3 (C-13″); 129.7 (C-4); 128.7 (C-3 and C-5); 128.2 (C-2 and C-6); 124.3 (C-7″); 123.6 (C-12″); 122.1 (C-8); 118.9 (C-2″); 118.7 (C-1′); 106.1 (C-5′); 100.3 (C-3′); 65.6 (C-1″); 39.7 (C-5″); 39.6 (C-10″); 26.2 (C-11″ and C-6″); 25.7 (C-15″); 17.7 (C-14″); 16.7 (C-4″); 16.0 (C-9″). MS: M + H ion *m/z* 445.6162 (calculated for C_30_H_36_O_3_: 444.605).

### 3.5. Oomycete Isolate and Culture Condition

In this study, the strains of *S. parasitica* and *S. australis* were used in all experiments. These strains were isolated from a naturally infected salmon (*Salmo salar*) [34] and was maintained on yeast dextrose agar medium at 4 °C [35]. The inoculum of the pathogen was grown on Saboraud dextrose agar medium at 18 °C for three days [36]. Molecular characterization and identification of strains of *S. parasitica* and *S. australis* was carried out according to the method detailed elsewhere [37].

### 3.6. Microwell Enumeration Method Biological Assays

This method is based on inoculating water samples, and experimental conditions have been detailed elsewhere [38].

### 3.7. Determination of Minimum Inhibitory Concentration (MIC)

The antioomycete activities of all tested compounds were evaluated using the dilution test at final concentrations of 3.125, 6.25, 12.5, 25.0, 75.0, 100.0, 125.0, 150.0 and 200.0 µg/L in Griffin's sporulation medium [39]. Bronopol was used as the positive control, whereas 1% solution of EtOH/tween 20 was considered as the negative control. Experimental conditions have been detailed elsewhere [37].

### 3.8. Spores Germination Inhibition Test

Spores from the culture on PDA plates were taken and suspensions of spores were made separately with different compounds [40]. The minimum oomyeticidal concentration (MOC) was defined previously [39]. Experimental conditions have been detailed elsewhere [37].

### 3.9. Mycelial Growth Inhibition Test

The anti-saprolegnia activities of all tested compounds were evaluated using the radial growth test at final concentrations of 200 µg/L in PDA medium. The growth inhibition rate will be calculated from mean values as:

**%IR = 100 (x − y)/(x − z)** (1)

where **IR** is the growth inhibition rate; **x**, the mycelial growth in control; **y**, the mycelia growth in sample; and **z**, the average diameter of the rapeseeds. Experimental conditions have been detailed elsewhere [37].

### 3.10. Measurement of Cellular Leakage

Cell leakage were measured in order to determine the effectiveness of compounds **1**–**11** on membrane integrity. This method was assessed according to Lunde [41].

### 3.11. Statistical Analysis

The data was reported following a standard method [37].

## 4. Conclusions

The results of this research evidence how the presence of a lipophilic alkyloxy chain modifies biological activity; the data so far has suggested that oxyalkylated chalcones may represent a new frontier and a challenge for the development of novel anti-oomycete compounds against *Saprolegnia* sp. in the near future. There appear to be no published studies investigating the effectiveness of 2′,4′-dihydroxychalcone (**1**) or its synthetic analogs **2**–**11** in terms of combating *Saprolegnia* infection. The natural compounds proved to be more effective at inhibiting *Saprolegnia* growth in vitro than ketoconazol or bronopol.

## Figures and Tables

**Table 1:** Minimum inhibitory concentrations (MIC) and damage values of oxyalkylchalcones **1**–**11** against *Saprolegnia* species.

Compounds	MIC (µg/mL)		Damage (%) ^a^	
	*S. parasitica*	*S. australis*	*S. parasitica*	*S. australis*
1	12.5	6.25	100	100
2	>200	200	0	0
3	200	175	16	20
4	>200	>200	10	15
5	175	150	28	30
6	>200	>200	0	0
7	200	175	28	30
8	>200	200	0	0
9	200	175	10	15
10	200	175	12	17
11	150	125	30	32
Bronopol	175	150	30	35
Safrole	175	150	30	35
Eugenol	150	150	35	38
Fluconazole	>200	>200	Nd	Nd
Ketoconazole	75	50	Nd	Nd
Sodium Dodecyl Sulfate	-	-	100	100

Nd: Not determined; ^a^ Damage produced by compounds **1**–**11** compared to the damaged produced by the Sodium Dodecyl Sulfate (SDS). SDS was utilized at a final concentration of 2% that produces a 100% of cell lysis. The assay was performed in duplicates.

**Table 2:** Minimum oomycidal concentrations (MOC) values ^a^ of oxyalkylchalcones **1**–**11** against mycelium at 48 h.

Compounds	MOC (µg/mL)	
	*S. parasitica*	*S. australis*
1	12.5	12.5
2	200	200
3	200	175
4	>200	>200
5	175	150
6	>200	>200
7	175	175
8	>200	>200
9	>200	200
10	200	175
11	125	125
Bronopol	>200	175
Safrole	>200	200
Eugenol	>200	175
Fluconazole	>200	>200
Ketoconazole	100	75

^a^ Each value represents the mean ± SD of three experiments, performed in quadruplicate.

**Table 3:** Mycelial Growth Inhibition values of oxyalkylchalcones **1**–**11** against *Saprolegnia* spp. at 48 h.

Compounds (200 µg/mL)	MIG (%)	
	*S. parasitica*	*S. australis*
1	100	100
2	32	35
3	33	36
4	0	0
5	35	38
6	0	0
7	30	33
8	0	0
9	0	0
10	10	13
11	50	55
Bronopol	0	33
